# Dielectrophoretic Microfluidic Designs for Precision Cell Enrichments and Highly Viable Label-Free Bacteria Recovery from Blood

**DOI:** 10.3390/mi16020236

**Published:** 2025-02-19

**Authors:** Dean E. Thomas, Kyle S. Kinskie, Kyle M. Brown, Lisa A. Flanagan, Rafael V. Davalos, Alexandra R. Hyler

**Affiliations:** 1 CytoRecovery, Inc., Blacksburg, VA 24060, USA; dthomas@cytorecovery.com (D.E.T.); kkinskie@cytorecovery.com (K.S.K.); kbrown@cytorecovery.com (K.M.B.); 2 Departments of Neurology, Biomedical Engineering, and Anatomy & Neurobiology, University of California Irvine, Irvine, CA 92697, USA; lisa.flanagan@uci.edu; 3 Wallace H. Coulter Department of Biomedical Engineering, Georgia Tech-Emory University, Atlanta, GA 30318, USA; rafael.davalos@bme.gatech.edu

**Keywords:** microfluidics, cell sorting, cell recovery, dielectrophoresis, commercialization, bacteremia, sepsis, bacteria, whole human blood

## Abstract

Conducting detailed cellular analysis of complex biological samples poses challenges in cell sorting and recovery for downstream analysis. Label-free microfluidics provide a promising solution for these complex applications. In this work, we investigate particle manipulation on two label-free microdevice designs using cDEP to enrich *E. coli* from whole human blood to mimic infection workflows. *E. coli* is still a growing source of bacteremia, sepsis, and other infections in modern countries, affecting millions of patients globally. The two microfluidic designs were evaluated for throughput, scaling, precision targeting, and high-viability recovery. While CytoChip D had the potential for higher throughput, given its continuous method of DEP-based sorting to accommodate larger clinical samples like a 10 mL blood draw, it could not effectively recover the bacteria. CytoChip B achieved a high-purity recovery of over 98% of bacteria from whole human blood, even in concentrations on the order of <100 CFU/mL, demonstrating the feasibility of processing and recovering ultra-low concentrations of bacteria for downstream analysis, culture, and drug testing. Future work will aim to scale CytoChip B for larger volume throughput while still achieving high bacteria recovery.

## 1. Introduction

Understanding subpopulations of complex biological samples is critical to biology, chemistry, and healthcare research. However, conducting detailed analysis at the cellular, particle, virus, and bacteria levels poses significant challenges, especially if researchers aim to continue studying these subpopulations after manipulation from the bulk sample. Microtechnology for cell manipulation and analysis has emerged as an option for improved cell and micro-level recovery of various biological samples. These devices can be particularly useful when label-free cell sorting solutions are desired in certain biomedical and life science research applications to maintain the native characteristics of the cells and particles. These label-free applications often arise where markers are either not well established or can alter particle behavior, such as the case of microbiomes [1,2] and stem cells, respectively [3,4,5,6]. Label-based sorting can typically only be used with fixed cells [7,8]. In the case of many cell types, including neurons, fixation of cells is not desired since it causes death, which limits the sample from being used further after sorting [9,10,11]. Markers cannot be effectively used with dynamically changing cells like in pre- and post-clinical treatment samples [9,12,13] and in cases where centralized equipment such as flow cytometers may not accommodate contaminating particles like bacteria and viruses [14,15]. Even relatively passive methods such as centrifugation can prove challenging for cell viability after processing, rendering the sample no longer usable for additional study [16,17]. Further, manipulating and recovering cells without labels can provide improvement in downstream analysis like PCR, single-cell sequencing, culture, and drug toxicity studies.

One such application involves targeting and enriching bacteria, such as isolating the cancer microbiome from a tumor or enriching bacteria from blood. This process is challenging because the particles vary significantly in size, spanning an order of magnitude [18]. Additionally, traditional fluorescent antibodies are unreliable [19,20], centrifugation is difficult [18], and shared equipment cannot be used due to the risk of contamination [14]. However, enriching and recovering these microbial particles from other cells is critical to downstream analysis like PCR, colony culture, and drug treatment analysis. Here, a microdevice must precisely target and manipulate small (usually ~1–2 μm) particles from larger cells (usually ~6–8 μm), often in ultra-low concentrations (e.g., 1 bacterium per >10^6^ cells). This requires balancing volumetric sample throughput with precision cell targeting to capture the rare events in large volumes [18,21,22]. In these applications, the experimental goal might be to effectively target and recover the rare bacteria cells for downstream PCR identification, analysis, and antimicrobial resistance detection. In contrast, in another application, sorting subpopulations of stem cells may not require consideration of size scaling since the cells are all the same order of magnitude, but the device must provide gentle, targeted enrichments where labels are ineffective due to cross-staining [23,24] and can further cause cells to begin differentiating or changing behavior [25,26]. Here, the experimental or healthcare goal might not be high-purity sorting but rather high-viability recovery for downstream analysis like single-cell sequencing, culture, and stem cell therapy development.

Thus, there are numerous microdevice designs given the complexity of biological sample inputs and the goals of particle enrichment, manipulation, and recovery. One method for label-free cell manipulation in microdevices is dielectrophoresis (DEP). DEP manipulates, sorts, and recovers cells and particles based on physical morphology and polarizability features like membrane capacitance, cytoplasm conductivity, cell size, and cell shape [27]. DEP has been used to analyze, sort, enrich, and recover [28,29] cancer stem cells [30,31,32,33,34], chemo-resistant cells [35], mesenchymal stem cells [36,37], neural stem cells [38,39,40], bacteria [41,42,43,44], live cells [45], proteins [46,47,48,49], organelles [50], extracellular vesicles [51,52,53,54], red and white blood cells [55,56,57,58,59,60], viruses [61], nanoparticles [62,63], and DNA [29,47,64], demonstrating its versatility across size scales and for precision particle manipulation and recovery. In DEP-based sorting devices, there are common features of electrodes that generate the non-uniform electric field and a sample channel for flowing and manipulating the biological sample. Contactless DEP (cDEP) takes DEP a step further by separating the biological sample completely with a thin ~10 μm membrane from the electrodes present in the device, hence the contactless name. This design better insulates samples from the electrodes, reducing Joule heating and better preserving cells’ native phenotypes [55,65]. This membrane layer allows the electrical field gradient to pass but buffers cell and particle samples to remain physically isolated [65,66,67,68,69]. As shown in the diversity of device designs in previous works, one singular DEP-based microdevice for all these applications or crossovers in particles and cells is not feasible to accommodate the varying experimental goals, sample inputs, and manufacturing requirements. For enriching bacteria, others have demonstrated feasibility using impedance cytometry [70,71,72], electro-photonic traps [73], acoustophoresis [74], or microbalances [75] on microfluidic devices, demonstrating valuable feasibility in a variety of application spaces. Other label-free sorting microdevice designs exist, such as deterministic lateral displacement (DLD) or inertial focusing, but these methods often suffer from a lack of sorting purity and the inability to sort across size scales since they rely heavily on size-based physics [76,77,78,79,80,81].

In this work, we investigate particle manipulation on two label-free microdevice designs using cDEP to enrich *Escherichia coli* (*E. coli*) from whole human blood to mimic infection workflows. *E. coli* is still a growing source of bacteremia, sepsis, and other infections in modern countries and impacts 47–50 million patients globally each year [82,83,84,85,86]. Strategies to faster isolate and identify the presence of the bacteria remain important to combat deadly hospital-acquired infections, which account for one in three deaths [87,88,89,90]. Compounding this challenge is the growing antimicrobial resistance globally, contributing to one million patient deaths in 2019 alone [91,92,93,94]. Here, the goal was to enrich very low concentrations of bacteria simulating bacteremia and sepsis, on the order of <100 colony forming units (CFU) per mL of whole human blood, with high enrichment efficiency and recovery of the bacteria to mimic bloodstream infection detection and sample prep for downstream analysis. In particular, the goal was to recover >90% of the bacteria present in whole blood while aiming to scale the high-purity enrichment to standard clinical blood samples of ~10 mL in volume. Experiments investigated a batch-based sorting device, CytoChip^TM^ B, and a continuous sorting device, CytoChip D, on the CytoR1^TM^ platform. The CytoR1 provides an integrated benchtop system with swappable, plug-and-play microtechnology devices deploying DEP for label-free sorting. Enrichment and recovery of *E. coli* from diluted whole human blood was evaluated with downstream plating, fluorescent microscopy, and colony counting. We ultimately evaluated the two CytoChip device designs in their ability to achieve the clinically relevant experimental goals of precision bacteria enrichment, highly viable bacteria recovery, and scaling for larger sample volume throughput starting from whole blood.

## 2. Materials and Methods

### 2.1. Device Geometry and Manufacturing

**Figure 1:**
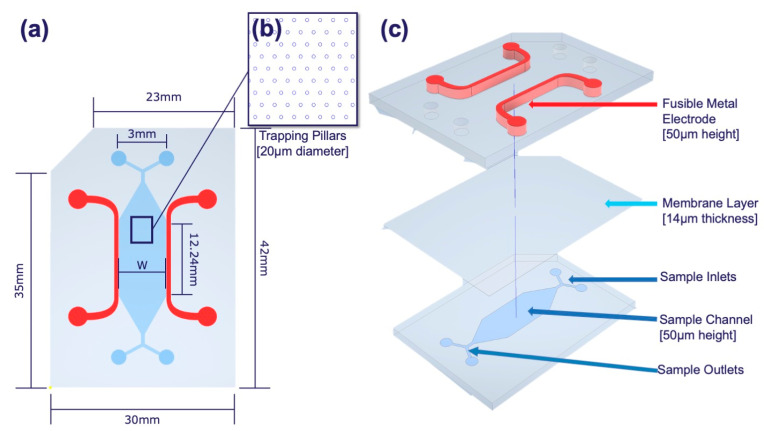
CytoChip B geometry and design features. (**a**) In this work, the width between the electrodes, w, was held constant at 3.4 mm. (**b**) Close-up of the pillars located in the sample channel and (**c**) the three-layer stack up.

CytoChip B (CytoRecovery, Blacksburg, VA, USA) was assembled using the processes previously described and with the geometry shown in Figure 1a–c [95,96]. Briefly, devices were designed in AutoCAD 2024 (Autodesk, San Francisco, CA, USA) and fabricated using standard photolithography to pattern silicon wafer molds. Deep reactive ion etching (DRIE) was performed by the University of Florida’s Nanoscale Research Facility (NRF) to produce the main channel and electrode channel layers on separate silicon wafers (University Wafer, South Boston, MA, USA) to a height of 50 μm. Etched wafers were treated with trichloro(1H,1H,2H,2H-perfluorooctyl) silane (Thermo Fisher Scientific, Waltham, MA, USA) following fabrication for easier polymer liftoff. Dow Sylgard 184 (Dow Chemicals, Midland, MI, USA), hereafter referred to as polydimethylsiloxane (PDMS), liquid base, and curing agent were combined in a 10:1 ratio and mixed for 8 min at 1000 rpm (Caframo Petite Stirrer, Alaveta, Branchburg, NJ, USA). Degassed polymer was poured onto the silicon master wafers at specific weights to control for thickness and create electrode and sample channel layers, then cured at 110 °C for 30 min. To make the membrane layers, a 5:1 ratio of polymer base to curing agent was mixed as above, and the polymer was statically spun (Model WS-650Mz-23NPPB, Laurell Technologies, Lansdale, PA, USA) at 4000 rpm onto a blank silicon wafer to obtain a 14 μm membrane thickness. The electrode layer was first bonded to the membrane layer using 1 min of air plasma at 0.6 Torr (Harrick Plasma, Ithaca, NY, USA) treatment and subsequently allowed to sit overnight at room temperature. Then, channel layers were bonded to the electrode–membrane layer using another air plasma treatment with guiding visual alignment marks. Fully assembled devices were allowed to sit overnight. Finally, electrode channels were filled with a fusible metal alloy (Thermo Fisher Scientific).

**Figure 2:**
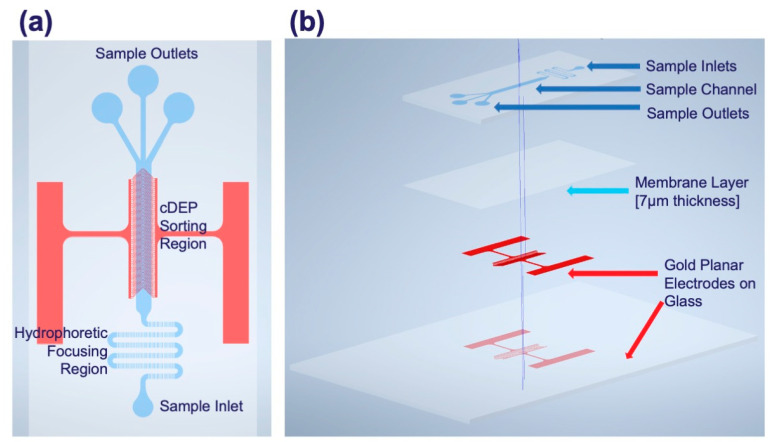
CytoChip D geometry and design features. (**a**) depicts top view of assembled, multi-layer device while (**b**) depicts the multi-layer channel, membrane, and electrode stack up. In this work, the total channel height was initially 70 μm (30 μm channel A layer, 40 μm channel B layer). Subsequent testing varied channel A from 20 to 50 µm and channel B from 30 to 50 µm in height. All other geometry features remained consistent, including hydrophoretic channel width (500 μm), electrode number (120), electrode spacing (65 μm), electrode width (35 μm), sorting channel width (1500 μm), and sorting channel length (13,200 μm).

CytoChip D (CytoRecovery) was manufactured as previously described with the geometries shown in Figure 2a,b below [38,97]. Briefly, devices were designed in AutoCAD (Autodesk) and fabricated using standard photolithography to pattern silicon wafer molds. Multi-layered SU-8 2025 (Kayaku, Tokyo, Japan) fabrication was conducted at the UF’s NRF to various final heights of the combined layers A and B to form the main channel. Etched wafers were treated with trichloro(1H,1H,2H,2H-perfluorooctyl) silane (Thermo Fisher Scientific) following fabrication for easier polymer liftoff. PDMS liquid base and curing agent were combined in a 10:1 ratio and assembled as described above. Degassed polymer was poured onto the silicon master wafer at specific weights to control for thickness and create sample channel layers and cured at 110 °C for 40 min. Glass electrode layers were manufactured at UF’s NRF using electron-beam physical vapor deposition or sputtering of titanium (200 Å) and gold (1000 Å). To make the membrane layers, a 5:1 ratio of polymer base to curing agent was mixed as above and then statically spun directly onto the glass electrode layers at 8000 rpm (Model WS-650Mz-23NPPB, Laurell Technologies). The electrode and membrane layers were then cured for 15–20 min at 90 °C. Finally, the electrode–membrane layer was visually aligned under a microscope and bonded to the main channel layer after 1 min of air plasma treatment (Harrick Plasma). Fully assembled devices were heated for 1 min at 80 °C and then allowed to sit at room temperature overnight before experimental testing.

Both CytoChip B and D employ DEP-based sorting as previously modeled and described [38,96]. In this work, the field gradients were modeled in COMSOL Multiphysics’ v6.1 (COMSOL, Inc., Burlingotn, MA, USA) electric currents module. A frequency domain sweeping 10 kHz to 15 MHz was input with an extra fine physics-controlled mesh to output the field strength. The conductivity and permittivity for all materials used are shown in Table 1 below. The respective field strengths were estimated to be 1.1 × 10^16^ V/m in CytoChip B and 4.4 × 10^18^ V/cm in CytoChip D.

### 2.2. Biological Sample Preparation

*E. coli* bacteria expressing green fluorescent protein (GFP) were thawed from frozen aliquots and cultured in suspension as recommended by the manufacturer (ATCC (Manassas, VA, USA) 25922GFP). Lysogeny Broth (LB) (Sigma-Aldrich, St. Louis, MO, USA) was prepared using manufacturer protocols, autoclaved for 15 min, and allowed to fully cool prior to experimental use. To the LB broth, a final concentration of 100 µg/mL ampicillin (Millipore Sigma, Burlington, MA, USA) was added. In summary, one frozen aliquot of ~1 × 10^6^ bacteria/mL was thawed rapidly from −80 °C. From the thawed sample aliquot, 100 µL of bacteria was suspended in 5 mL warmed LB broth in a sterile, vented tube (Bio-One Round Bottom Polypropylene Culture Tube Vented Stopper, Greiner, Kremsmünster, Austria) using aseptic techniques. The culture tube was grown at 37 °C, 150 rpm for 2 h to obtain a revival culture. After 2 h, 100 µL of revival culture was transferred to a fresh tube containing 8 mL of warmed LB broth and grown into an OD_600_ (test culture) after approximately an additional 80 min at 37 °C and 150 rpm. OD values were converted from measurements taken on a calibrated McFarland reader (DensiCHECK Plus, Biomerieux, Marcy-l’Étoile, France).

Healthy, human donor whole blood was obtained from Precision for Medicine (Mansfield, MA, USA) in ethylenediamine tetra acetic acid (EDTA) sterile tubes. Precision for Medicine is an FDA-registered blood establishment that follows all ethical requirements for donors in terms of human subject compliance and obtaining informed consent for their blood products for research use. Initially, frozen blood samples were tested in the feasibility studies. Upon workflow optimization, fresh blood samples were used, which more closely represents a clinical laboratory scenario. Frozen blood was stored at −20 °C and used immediately upon thawing. All fresh blood was stored at 4 °C until experimental use and was not used beyond 3 days since healthy donor donation. The fresh blood was warmed to room temperature before testing to standardize conductivity readings.

CytoBuffer^TM^ formulations were tested for washing and utility in experimentation to decrease sample coagulation, sustain bacteria viability, and reduce sample conductivity to allow DEP-based sorting [98]. CytoBuffers H and I were used to wash the sample. CytoBuffer I contains Triton-X 100 and anticoagulant acid citrate dextrose solution A (ACD-A) for lysing the red blood cells and preventing the platelets from coagulating, respectively, whereas CytoBuffer H contains only the ACD-A additive and sucrose. CytoBuffers H and I were measured to be <70 µS/cm (LAQUAtwin-EC-11, Horiba, Kyoto, Japan) upon mixing and were stored at 4 °C for up to 14 days.

Whole blood was rocked continuously on a small 3D rotator (Thermo Fisher Scientific). Following a minimum of 20 min of rocking and thawing at room temperature, whole blood was processed for final dilution into CytoBuffer J, which contains the same ingredients as CytoBuffer H but also includes an anti-fouling agent to reduce cell fouling. First, 500 µL whole blood was washed into 500 µL CytoBuffer I and centrifuged at 6000× *g* for 3 min (accuSpin micro 17, Thermo Fisher Scientific). Supernatant was removed, and 900 µL fresh CytoBuffer H was added to gently resuspend the whole blood and centrifuged again at the conditions above. Supernatant was again removed from the whole blood, which was resuspended a final time into fresh CytoBuffer J to desired concentrations. After two washes, diluted whole blood was counted (Countess 3FL, Thermo Fisher Scientific), and the suspension was adjusted to a final concentration of 1–2 × 10^6^ cells/mL. When desired, bacteria were then added into the diluted blood sample to desired CFU/mL concentrations. Validation of the CFU/mL dilution of bacteria into the blood was conducted and confirmed first by independently processing bacteria only into CytoBuffers, followed by plating the resulting samples to confirm colony counts. After processing into CytoBuffer, bacteria were streaked onto LB agar plates with 100 µg/mL ampicillin (Thermo Fisher Scientific) and incubated at 37 °C with 5% CO_2_ (Heracell Vios 160i, Thermo Fisher Scientific). After 16+ h, colonies were counted for viability and concentration confirmation (*n* = 3). For all experiments, the final conductivity of the input sample and blank CytoBuffer J were measured to be <100 µS/cm. Two 1 mL syringes (NORM-JECT, Henke Sass Wolf, Tuttlingen, Germany) were loaded with the prepared sample in CytoBuffer J and blank CytoBuffer J, respectively.

Initially, diluted whole human blood or *E. coli* bacteria were independently loaded onto the CytoChip B and D designs to investigate their dielectrophoretic responses. Highly concentrated samples of bacteria and whole blood (1000+ CFU/mL and 2 × 10^6^ cells/mL, respectively) in CytoBuffer J were used for response characterization on the two investigated microdevice designs. Following characterization, *E. coli* bacteria were contrived into the diluted whole human blood sample. A final target bacteria concentration of 25 CFU/mL in 100 µL of diluted whole blood sample in CytoBuffer J was used to obtain device design performance.

### 2.3. Experimental Procedures on CytoR1 Platform and CytoChip Performance Evaluation

**Figure 3:**
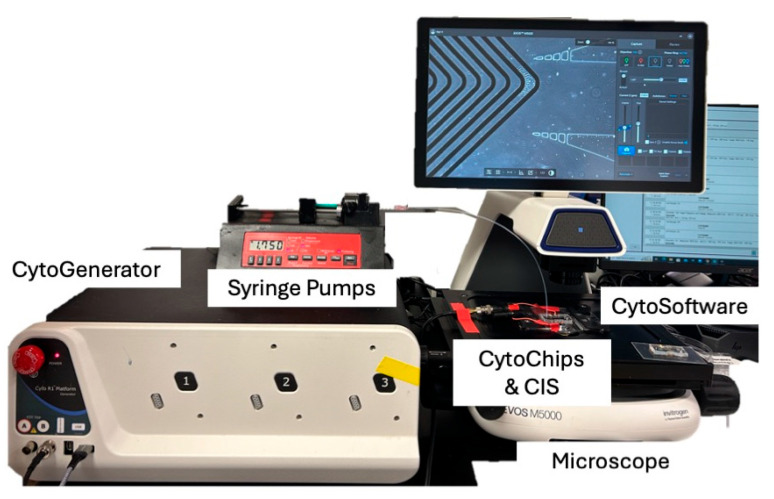
CytoR1 platform setup for experimental testing of CytoChips B and D for label-free, cDEP enrichment of *E. coli* from whole human blood.

The CytoR1 platform is a benchtop instrument for integrated cell enrichment and recovery, as shown in Figure 3. The platform consists of a Chip Interfacing System (CIS) to easily plug and play various microdevice designs electronically and fluidically (D6422, CytoRecovery). Additionally, the CytoGenerator^TM^ (CytoRecovery), a wideband power amplifier, produces 5 kHz–15 MHz and 2 Vpp–640 Vpp for dielectrophoretic field gradient generation [99,100]. Two syringe pumps (New Era 1000-US) control the sample, and blank CytoBuffer entry and all the hardware are controlled by CytoSoftware^TM^ v1.1.5.4 (CytoRecovery, Blacksburg, VA, USA). For this work, experiments were visualized using a fluorescent microscope with GFP and Texas Red filter light cubes (Evos M5000, Thermo Fisher Scientific).

Once the bacteria were added into the diluted blood sample and blank CytoBuffer reagent were loaded into syringes as above, they were each connected to a 20-gauge needle (SANANTS 0.5-inch, Amazon, China) and 1/32-inch inner diameter PTFE tubing (Master Flex, Gelsenkirchen, Germany). CytoChip priming was conducted using standard 70% ethanol (Thermo Fisher Scientific) loaded into a 2 mL syringe (Nevershare, Exchange Supplies, Dorchester, UK) connected via the same needle and tubing as samples to remove all bubbles from the microdevices. Once primed and all bubbles removed, blank CytoBuffer J was flushed through the chips to ensure no toxicity to the samples. For CytoChip B, flow rates were varied from 1 to 5 μL/min for the sample and 0.1 μL/min for blank CytoBuffer. For CytoChip D, flow rates were varied from 1 to 20 μL/min for the sample.

Once loaded onto the CytoR1 platform using one of the two CytoChip designs, *E. coli* bacteria were enriched from the diluted whole human blood. The CytoGenerator was tuned to a variety of parameters to determine a high-purity sort and recovery of the bacteria, where the blood cells were considered waste for the purpose of detecting potential bloodstream infections. Frequency (10 kHz–15 MHz) and voltage (10 Vpp–640 Vpp) parameters were swept and explored to locate where >90% of *E. coli* was experiencing DEP, which was determined to be effective trapping on each chip design. For CytoChip B, replicates were batched and collected after 30–60 min of run time. A wash step using blank CytoBuffer J at 5 μL/min was conducted between waste and target bacteria collection to clear the device of any remaining sample and ensure high recovery. For CytoChip D, 20–60 min of continuous sample processing was considered one replicate.

Recovered bacteria from the waste and collection streams were initially plated into 96-well flat bottom plates (GenClone, Genesee Scientific, El Cajon, CA, USA) and incubated as above. After 16+ h of post-sorting growth, wells were imaged on the scope above. As workflows were optimized, recovered bacteria from the waste and collection were both streaked onto LB agar plates with 100 µg/mL ampicillin (Thermo Fisher Scientific) and incubated as above.

### 2.4. Statistical Analysis

All counts shown were conducted in triplicate, with final counts shown as the average ± standard error of the mean (SEM).

## 3. Results

### 3.1. Sample Preparation Validation

*E. coli* was plated, grown, and counted after processing in CytoBuffer to achieve final concentrations of 1–1000 CFU/mL and a conductivity below 100 µS/cm. Samples <20 CFU/mL varied significantly in colony count (±100%) and so were considered unreliable given the losses of the sample during processing and transfer to the plates. Samples >200 CFU/mL resulted in too high of a confluency on the plates, so counts could not be obtained as colonies merged. *E. coli* concentrations of 20–200 CFU/mL were found to be consistent (±20%) and deemed repeatable and accurate for processing, experimentation, and subsequent plating and counting. These initial seeding experiments further validated that the bacteria grew as expected after the washing and processing steps into low conductivity CytoBuffers.

### 3.2. CytoChip B and D Initial Performance Evaluation

**Figure 4:**
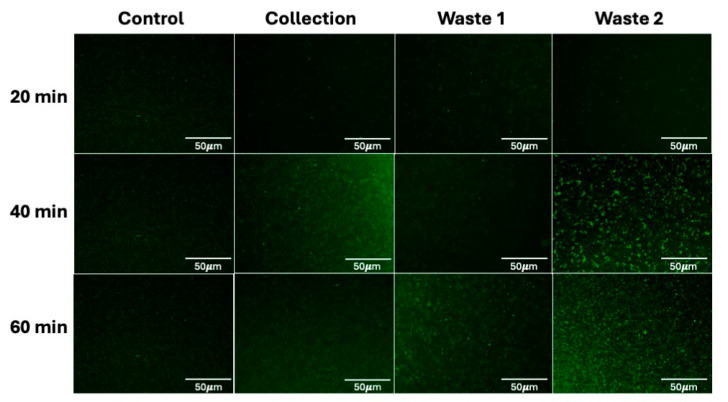
CytoChip D imaging results from preliminary *E. coli* from diluted human whole blood enrichment at 500 kHz and 578 Vpp. Here, GFP microscopy imaging shows just the bacteria recovery after 20, 40, and 60 min across the collection and waste channels. Initial results indicate a lot of the bacteria was not enriched and ended up in the waste stream, but it is also important to note that the control sample also appeared lower in concentration than anticipated. These results indicated alterations to the device geometry were needed for further optimization.

CytoChip D was deployed continuously such that the bacteria migrated (positive DEP) into the center outlet channel while the blood cells passed into the two outer waste channels (no DEP). Initially, this design was selected to afford flexibility in scaling to larger volumes that would be present in clinical samples, like 10 mL blood draws. When optimized, 500 kHz and 578 Vpp were used to achieve the best bacteria enrichment and recovery. Initially, failure was observed in CytoChip D’s performance because the bacteria and blood cells escaped the hydrophoretic focusing upstream of the DEP-based sorting, meaning the bacteria and cells were not actually experiencing DEP-based sorting but rather flowing directly through the device under drag. An example of this drag-dominated phenomenon is shown in Supplementary Video S1. The initial evaluation results of bacteria enrichment, plating, and microscopy imaging from diluted whole human blood on CytoChip D are shown in Figure 4.

**Figure 5:**
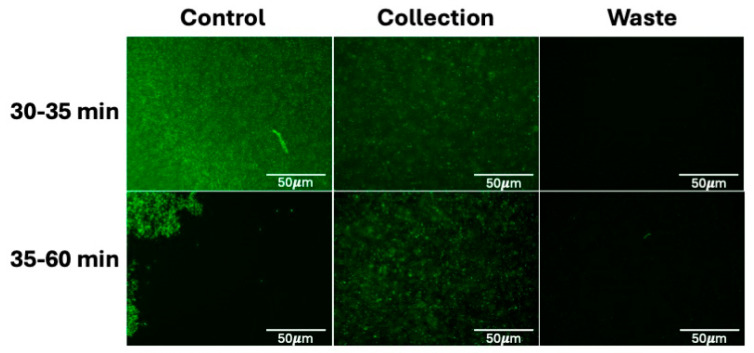
CytoChip B imaging results from preliminary enrichment of *E. coli* from diluted human whole blood at 400 kHz and 648 Vpp. Here, GFP imaging shows just the bacteria recovery after ~30 and ~60 min in the collection and waste batches. Initial results indicate enrichment from the control into the collection batch, driving the decision to continue with the device geometry and further optimize the workflow on CytoChip B in subsequent testing.

CytoChip B was deployed such that the bacteria were trapped (positive DEP) on the pillars while the blood cells passed through. When optimized, 400 kHz and 648 Vpp were determined to achieve the best bacteria enrichment and recovery, as shown in Supplementary Video S2. Using this workflow, the bacteria were effectively trapped at a high rate, but the flow rate was initially maximized at only 1–2 μL/min, meaning scaling to larger volume samples would require some innovative approaches. Regardless, CytoChip B showed improved enrichment over CytoChip D in initial experimentation. The initial experimental results of bacteria enrichment, plating, and imaging from diluted whole human blood on CytoChip B are shown in Figure 5.

### 3.3. CytoChips Optimization for High-Purity Bacteria Enrichment and Recovery

Following initial experimental evaluation, both CytoChip workflows and microfeature geometries were further developed to improve performance. For CytoChip B, the focus was on improving the volumetric throughput since this design is limited by batching times and speeds. For CytoChip D, the device geometry was altered to focus on improving the enrichment efficiency and ensuring the bacteria and blood cells could be hydrophoretically focused for DEP-based sorting downstream. Lower concentrations (30,000 and 1000 CFU/mL after dilution) were tested on the evolved workflows on both CytoChip B and D.

**Figure 6:**
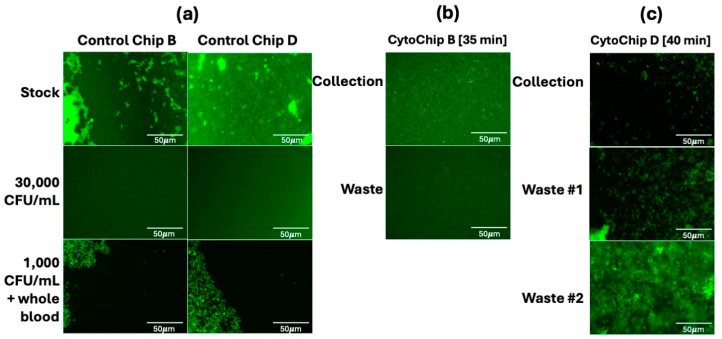
CytoChips microscopy imaging results from optimized workflows of *E. coli* from diluted human whole blood enrichment. (**a**) depicts the control stock and two dilutions of *E. coli* in whole blood. (**b**) demonstrates the enrichment of *E. coli* into the collection batch with few bacteria in the waste batch in CytoChip B. (**c**) illustrates CytoChip D still contains a lot of bacteria that are leaking into the waste channels and that little are recovered in the collection channel despite altering the channel height to attempt to better focus the sample upstream of the DEP sorting region.

First, CytoChip B flow rates were increased as much as possible to continue building toward higher volumetric throughput. A maximum processing speed of 3.3 μL/min was obtained with continued, high enrichment of the bacteria in the collection batch, as shown in Figure 6b. Additionally, low concentrations of bacteria were found in the waste batch.

To combat the lack of focusing in CytoChip D, altered geometries were made with 60–70 µm total heights to improve the hydrophoretic focusing of the bacteria and blood cells upstream of the DEP sorting region. Specifically, channel A was varied from 20 to 50 µm, and channel B was varied from 30 to 50 µm in height. However, these altered designs still resulted in low enrichment, with high concentrations of bacteria ending up in the waste streams over the collection stream, as shown in Figure 6c.

### 3.4. Toward a Clinically Relevant Evaluation of the Optimized CytoChip B Workflow

**Figure 7:**
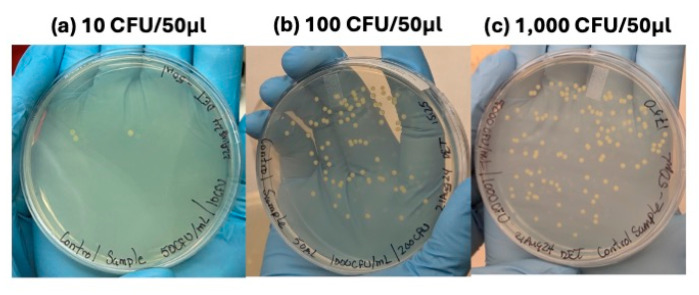
Demonstration of *E. coli* dilution at various orders of magnitude, including (**a**) ~10 CFU/50 µL, (**b**) 100 CFU/50 µL, and (**c**) 1000 CFU/50 µL. Cultures were streaked, plated, and incubated for 16+ h of growth.

Given the initial performance evaluation, workflow optimization, and design changes, CytoChip B was shown to have a better preliminary enrichment for bacteria from whole blood. To mimic a more clinically relevant sample, a <50 CFU/mL concentration of bacteria was in the diluted whole blood with 100 µL of total sample volume processed. To validate the appropriate dilution schemes and plating procedures, controls were first obtained for several orders of magnitude of bacteria concentration, as shown in Figure 7. During this process, it was observed that concentrations ≤10 CFU/mL had high variability (±100%) due to the losses in processing and subsequent plating. Concentrations of ≥20 CFU/mL were found to be significantly less variable (±30%).

**Figure 8:**
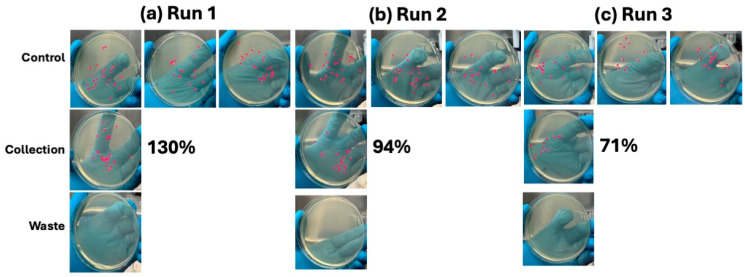
Results of the optimized CytoChip B workflow using 3.3 µL/min flow rate resulting in 30 min to batch a 100 µL sample containing 25 CFU bacteria. For each replicate run (**a**–**c**), multiple controls, the collection batch, and the waste batch were recovered, streaked, and grown for 16+ h. Red dots indicate counted colonies for data analysis, and the recovery percentage is taken relative to the average of the three controls for each experimental run.

**Figure 9:**
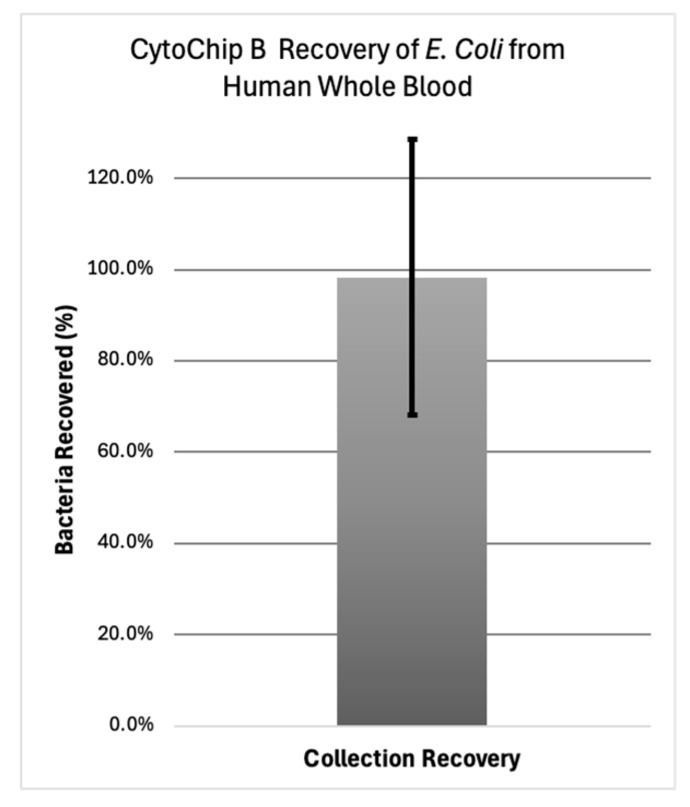
Optimized CytoChip B workflow achieved, on average, a 98.6% recovery of 25 CFU of *E. coli* in 100 µL diluted whole human blood. High variability in plating is known to be one challenge of colony counting correlations, which can cause higher standard errors in the mean [101].

To achieve the appropriate dilutions of bacteria in whole blood, optimized workflow enrichments were conducted on ~25 CFU/100 µL samples using the 3.3 µL/min max flow rate, resulting in 30 min to batch the full sample. Following enrichment, the waste batch and collection batch were streaked and plated to determine the recovery percentage and enrichment purity. As shown in Figure 8a–c, 21 ± 9 bacteria were recovered in the collection batch, with 0 ± 0 bacteria recovered in the waste also containing the blood cells. This equated to a 98.4% recovery efficiency of the bacteria on CytoChip B’s optimized workflow, as shown in Figure 9.

## 4. Discussion

The complexity of biological samples in healthcare and biotechnology applications can prove challenging when there is a desire to understand subpopulations of cells and particles and their relative interactions, behaviors, and patterns. In this study, we explored cDEP as a label-free method for enriching cells and particles from heterogeneous suspensions. Label-free sorting is highly valuable in workflows where the cells or particles are going to be further studied downstream in analyses like sequencing, PCR, culture, and drug toxicity testing. In this work, we aimed to enrich *E. coli* from whole human blood to target and recover the bacteria for downstream analysis, mimicking clinical workflows for sepsis and bloodstream infection detection. Of important note, spiked samples in healthy blood were used, which does not fully account for the expected clinical variability in patient-obtained samples, which will need to be further explored to validate this initial work. Other environmental factors and variables like temperature control, other bacteria strains, blood draw to experimental time, humidity control, and blood collection tube coatings could further alter these findings as complexity is increased. The experimental goals were complex, requiring a balance between high-viability recovery without labels and large volume throughput. Therefore, two DEP-based sorting microdevices, CytoChips B and D, were investigated for their unique advantages and disadvantages in enriching *E. coli* from healthy donor human whole blood. The two designs were evaluated for throughput, scaling, precision targeting, and high-viability recovery. While CytoChip D had the potential for higher throughput, given its continuous method of DEP-based sorting to accommodate larger clinical samples like a 10 mL blood draw, it could not effectively recover the bacteria. In CytoChip D, a significant sample was lost in the waste due to inadequate focus within the hydrophoretic region. Even with design alterations to improve this fluidic focusing upstream of the DEP sorting, CytoChip D underperformed in bacteria enrichment and recovery. Of note, CytoChip D was designed for mammalian cells and not for smaller cells such as bacteria, and additional design modifications may be necessary to target small cells [38,39]. Though it did not work in this particular application, CytoChip D affords many advantages in other applications like sorting stem, cancer, and immune cells. CytoChip B showed consistent, high-purity recovery of bacteria, even in concentrations on the order of <100 CFU/mL. CytoChip B achieved over 98% viable recovery of the *E. coli* while depleting the blood cells, demonstrating the feasibility of processing and recovering ultra-low concentrations of bacteria for downstream analysis, culture, and drug testing. While promising, scaling the precision recovery of CytoChip B for large-volume samples could prove challenging in moving toward clinical applicability. Future work will investigate parallelizing CytoChip B or lengthening the sample channel to provide larger volume throughputs. Additionally, integrating other label-free sorting designs, such as deterministic lateral displacement (DLD), could be investigated in combination with CytoChip B to balance bulk and higher throughput sorting upstream with precision enrichments downstream. If the 10 mL clinical sample can be depleted of the high concentration of red blood cells (~40% of 10 mL) using upstream DLD or lysis, the downstream processing of ~6 mL of concentrated bacteria in the remaining plasma and buffy coat could be achieved on a four-chamber array of CytoChip B (13.2 µL/min), bringing the processing time down to 7–8 h instead of the days currently required for blood smear cultures [22,102]. Other novel processing, chemical treatments, or combinatorial solutions will likely be required to obtain rapid bacteria enrichment on the desired clinical scale of minutes to hours. In conjunction with exploring hybrid microdevice solutions, it will be important to compare other traditional methodologies and current gold standards, such as flow cytometry and blood smear cultures, to validate head-to-head comparisons.

## Figures and Tables

**Table 1:** Materials properties used for COMSOL field strength simulations of CytoChips B and D.

Material	Geometry Element	Relative Permittivity (Unitless)	Electrical Conductivity (S/m)
PDMS	Main Channels and Membranes	2.7	3.45 × 10^−13^
Metal Alloy	CytoChip B Electrodes	1	1.92 × 10^4^
Gold	CytoChip D Electrodes	1	4.24 × 10^7^
CytoBuffer	Sample Fluid	80	1.0 × 10^−2^

## Data Availability

The data presented in this study are available upon request from the corresponding author due to privacy restrictions.

## Supplementary Materials

The following supporting information can be downloaded at: https://cloud.cytorecovery.com/s/EdxCfzo7oem4Ead (accessed on 6 February 2025), Video S1: CytoChip D Enrichment Example, Video S2: CytoChip B Enrichment Example. Video S1 Caption: Example video of E. coli and diluted whole human blood on CytoChip D. As observed, much of the cells and bacteria are not hydrophoretically focused to the outer walls of the device as designed, meaning the downstream DEP enrichment region is not performing as expected. Video S2 Caption: These two videos depict same sample being sorted. (a) The e.coli, the target, is at a concentration of 10CFU/mL, and is visualized using the green filter. (b) The whole blood sample is shown flowing past the same trap points and visualized in bright field.
